# ELDERLY CARE POLICY IN CHINA: ITS EVOLUTIONARY PATH AND IMPLICATIONS ON GOVERNANCE

**DOI:** 10.1093/geroni/igad104.3298

**Published:** 2023-12-21

**Authors:** Qingwei Wang, Yu Ma, Jiayu Chen

**Affiliations:** Xi’an Jiaotong-Liverpool University, Suzhou, Jiangsu, China (People’s Republic); University of Bristol, Bristol, England, United Kingdom; Xi’an Jiaotong-Liverpool University, Suzhou, Jiangsu, China (People’s Republic)

## Abstract

Over the years, the Chinese elderly care policy has undergone significant changes, leading to the systematic development of a comprehensive elderly care service system. This study explores the pivotal policies formulated by central policy-making bodies and examines the historical and current state of the elderly care system in China. The findings demonstrate a consistent alignment between the system’s evolutionary trajectory, the practical expectations of the population, and the Communist Party of China’s (CPC) objective of fostering a unified and balanced society. The study also highlights inherent issues concerning governance mechanisms and role-position within the system. Notably, we highlight the successful protection of older adults during the COVID-19 pandemic as an exemplary care of care service provision and draw lessons from international experience.

